# Impact of Non‐Anesthesiologist‐Administered Propofol Sedation for Outpatient Endoscopy in the Healthcare System

**DOI:** 10.1002/deo2.70151

**Published:** 2025-06-05

**Authors:** Francesco Vito Mandarino, Giorgia Gribaudo, Noemi Salmeri, Lorella Fanti, Alberto Barchi, Luca Massimino, Ernesto Fasulo, Giuseppe Dell'Anna, Francesco Azzolini, Edi Viale, Edoardo Vespa, Lorenzo Quario, Antonio Facciorusso, Lorenzo Fuccio, Lorenzo Giovanni Mantovani, Paolo Angelo Cortesi, Silvio Danese

**Affiliations:** ^1^ Department of Gastroenterology and Gastrointestinal Endoscopy IRCCS San Raffaele Hospital Milan Italy; ^2^ Research Centre on Public Health (CESP) University of Milan‐Bicocca Monza Italy; ^3^ Department of Medical and Surgical Sciences University of Bologna Bologna Italy; ^4^ Department of Clinical Sciences and Community Health University of Milan Milan Italy; ^5^ Department of Anaesthesiology IRCCS San Raffaele Hospital Milan Italy; ^6^ Gastroenterology Unit, University of Lecce Lecce Italy

**Keywords:** costs, endoscopy, non‐anesthesiologist‐administered propofol, propofol, sedation

## Abstract

**Introduction:**

Non‐anesthesiologist‐administered propofol (NAAP) sedation for outpatient endoscopy has proven to be safe. However, implementing NAAP in Western countries faces challenges, and propofol‐based sedation is still largely administered by anesthetists. For low‐risk patients, anesthesiologist‐administered propofol (AAP) could represent an avoidable waste of healthcare resources.

**Methods:**

This research consisted of two phases. The first is a retrospective study comparing NAAP and AAP for outpatient endoscopy at a tertiary center, with the primary outcome being the rate of adverse events (AEs). Propensity score matching was performed to balance baseline characteristics between the two groups. The second phase involved a budget impact model to assess the economic impact of using NAAP instead of AAP for low‐risk patients, both locally and nationally, between 2023 and 2025.

**Results:**

Between May 2019 and November 2021, 2721 patients undergoing esophagogastroduodenoscopies (EGDs; NAAP 2439 and AAP 282) and 2748 colonoscopies (NAAP 2491 and AAP 257) were enrolled. Overall, the AE rates were similar between the cohorts (esophagogastroduodenoscopies: NAAP 1.1% vs. AAP 0.8%, *p* = 0.81; colonoscopies: NAAP 1.8% vs. AAP 3.5%, *p* = 0.20). All NAAP‐related AEs were minor.

The budget impact model revealed that adopting NAAP instead of AAP would save €124,724,659 and 2223 working days for healthcare professionals for the Italian National Health System (NHS) between 2023 and 2025.

**Conclusion:**

NAAP has a comparable AE rate to AAP for low‐risk outpatient endoscopy. Implementing NAAP instead of AAP could save over €100 million and 2000 working days for the Italian NHS between 2023 and 2025. Wider adoption could improve healthcare resource allocation.

## 1 Introduction

Endoscopy is a key minimally invasive tool for diagnosing and treating gastrointestinal diseases. However, patient anxiety and fear can reduce their willingness to undergo the procedure and affect the endoscopist's performance [1, 2, 3]. Sedation during endoscopy can alleviate patient discomfort, improving examination quality.

Until recently, outpatient endoscopy involved minimal to moderate sedation with a benzodiazepine to minimize anxiety, and a narcotic analgesic to alleviate discomfort and pain [4]. Thanks to its pharmacokinetic properties, including rapid onset (30–45 s) and short duration of action (4–8 min), propofol has become the preferred sedative for outpatient endoscopy in recent years [4].

Large‐scale studies have shown that propofol, when administered by endoscopists under Non‐Anesthesiologist‐Administered Propofol (NAAP) sedation, has an excellent safety profile [5, 6]. However, the implementation of NAAP in Western countries faces challenges, and propofol‐based sedation for gastrointestinal endoscopy continues to be mainly administered by anesthesia professionals under the Anesthesiologist‐Administered Propofol (AAP) model.

Concerns over propofol use by non‐anesthetist providers are largely influenced by professional politics rather than evidence‐based data. In the absence of proven benefits, AAP for low‐risk patients may lead to unnecessary healthcare spending.

This study aims to demonstrate NAAP safety versus AAP and explore the potential economic impact of expanding NAAP from a local to a national scale.

## 2 Materials and Methods

This study is structured in two main phases. The first is a retrospective observational analysis comparing NAAP and AAP at a tertiary center (IRCCS San Raffaele Hospital, Milan, Italy) in low‐anesthesiological‐risk patients, defined as those with American Society of Anesthesiologists (ASA) scores 1 and 2 [7]. The second phase involves a budget impact model (BIM) to assess the economic impact of implementing NAAP instead of AAP for low‐risk patients at the institutional (IRCCS San Raffaele Hospital, Milan), local (Health Protection Agency of Milan – ATS Milan), regional (Lombardy), and national (Italian NHS) levels over 2023–2025.

### 2.1 Observational Study

The study was conducted in accordance with the Strengthening the Reporting of Observational Studies in Epidemiology guidelines [8].

From May 2019 to November 2021, endoscopic procedures at a tertiary center were recorded.

The inclusion criteria were ASA scores of 1 and 2, age > 18 years, and the ability to provide written informed consent. Outpatient interventional procedures were excluded (Table S1). At our institution, NAAP is used for all low‐risk outpatient procedures, while AAP is reserved for private, insurance‐funded, or self‐paid cases. Data for each patient were prospectively recorded in an electronic system (Endoxweb, Tesigroup, Italy) and later analyzed retrospectively.

The study was approved by the Ethics Committee of the IRCCS San Raffaele Hospital (Protocol REG_EGDS COLON, approval code: 79/12/2022).

All patients provided informed consent for the use of their data for scientific purposes.

#### 2.1.1 Endoscopy and Sedation Procedures

NAAP and AAP used a TCI pump (Terufusion‐TIVA/TCI TE372 Terumo Europe N.V., Leuven, Belgium or Diprifusor AstraZeneca, Macclesfield, UK), as previously described (Appendix S1) [9, 10]. All endoscopists performing NAAP had dedicated training in the use of propofol, per institutional protocols and international guidelines [7].

#### 2.1.2 Outcomes

The primary outcome was the rate of adverse events (AEs) in the NAAP versus AAP cohorts. Major AEs included Intensive Care Unit admission, resuscitation, intubation, or death. Minor AEs were defined as described previously (Appendix S2) [9].

Secondary outcomes included procedure duration and propofol dosage.

#### 2.1.3 Statistical Analysis

Quantitative variables were reported as means and standard deviations; qualitative variables as absolute values and percentages. Independent t‐tests compared continuous variables, and Chi‐squared tests were used for categorical variables.

Propensity score matching (PSM) was adopted to balance baseline differences between the NAAP and AAP groups (Appendix S3).

Statistical significance was set at *p* < 0.05. Analyses were conducted using STATA SE version 18 (StataCorp, College Station, TX, USA).

### 2.2 Budget Impact Model

In the second part of the study, a budget impact model (BIM) for 2023–2025 was developed.

BIM was designed in accordance with the 2020 guidelines of the Italian Medicines Agency [11] and the International Society for Pharmacoeconomics and Outcomes Research's Task Force on Good Research Practices for budget impact analysis [12].

The model compared two scenarios: the “AAP scenario” (scenario 1), where all low‐risk patients (ASA scores 1 and 2) undergo endoscopy under AAP, and the “NAAP scenario” (scenario 2), where all low‐risk patients opt for NAAP. It estimated per‐treated patient and total costs, calculating incremental costs of NAAP versus AAP over the 2023–2025 period and for each year. Additionally, drug costs and the staff workload, expressed in healthcare professional days, were assessed. Costs were expressed in euros (€).

#### 2.2.1 Model Input Data

The proportion of low‐risk patients undergoing endoscopy was estimated from IRCCS San Raffaele Hospital data in 2023 and applied to populations at ATS Milan and Lombardy [13]. Nationwide rates of low‐risk patients undergoing endoscopy were extrapolated based on the rates observed in Lombardy and applied to the Italian population. Data for the Italian population were sourced from ISTAT data for 2023 [14], with projections for 2024–2025 based on ISTAT's population growth trends [14].

Endoscopy costs for NAAP and AAP were sourced from IRCCS San Raffaele's Health Management and applied to regional and national levels, following the International Society for Pharmacoeconomics and Outcomes Research's Task Force on Good Research Practices for budget impact analysis guidelines [12, 15]. Costs included patient preparation, procedure, and post‐procedure monitoring (Table S2). Details of personnel workload and drug costs are shown in Tables S3 and S4.

#### 2.2.2 Sensitivity Analyses

Deterministic sensitivity analyses were conducted to assess the robustness of BIM results, by varying, within a ± 10% range, one of the following parameters: Italian population, rate of the population undergoing EGD or colonoscopy, and rate of the low‐risk population undergoing EGD or colonoscopy.

## 3 Results

### 3.1 Study Design

**FIGURE 1:**
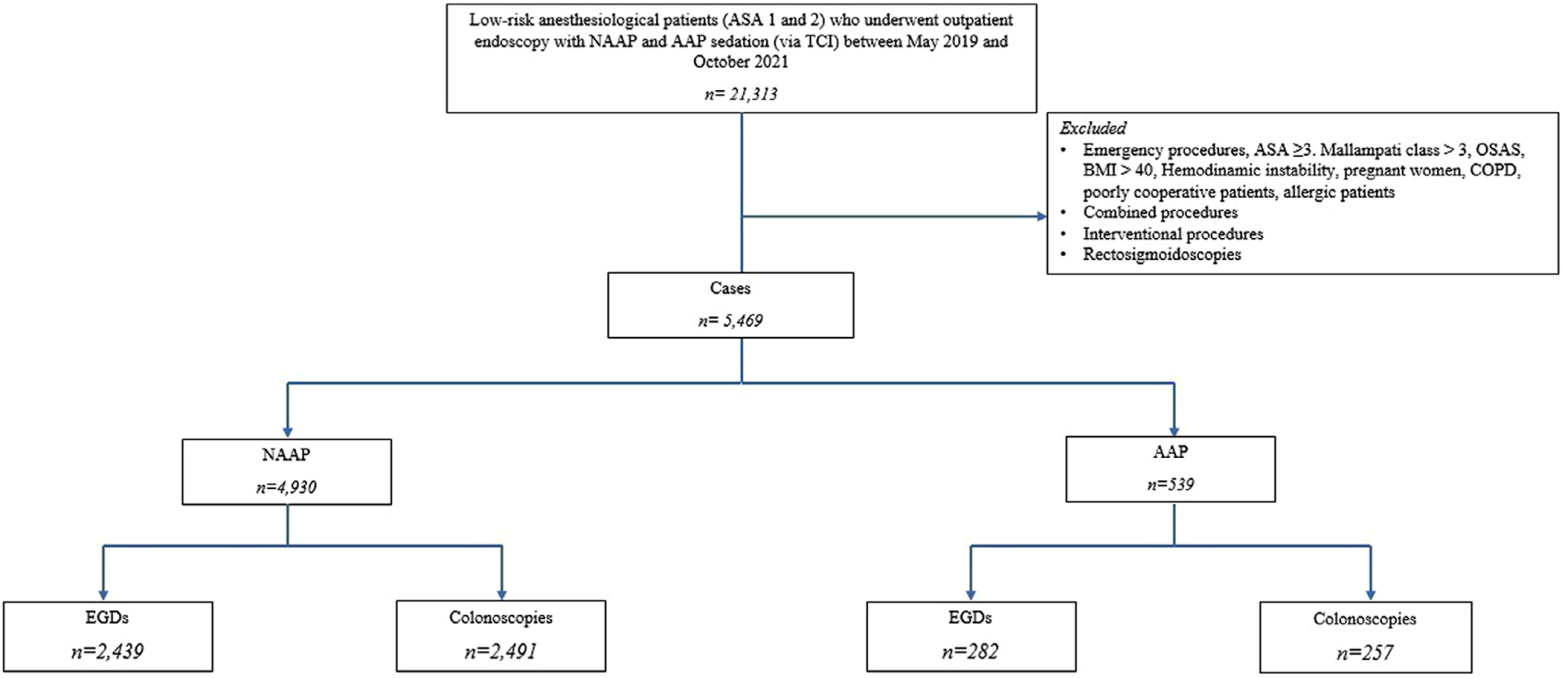
Study design: NAAP, non‐anesthesiologist‐administered propofol; AAP, anesthesiologist‐administered‐propofol; ASA, American Society of Anesthesiologists; OSAS, obstructive sleep apnea syndrome; BMI, body mass index; COPD, chronic obstructive pulmonary disease; EGD, esophagogastroduodenoscopy.

The study flow chart is shown in Figure 1.

Between May 2019 and October 2021, 21,313 patients underwent endoscopy at San Raffaele Hospital. After applying exclusion and inclusion criteria, 4930 NAAP and 539 AAP procedures were included.

### 3.2 Baseline Characteristics

#### 3.2.1 EGDs Cohort

Two thousand four hundred thirty‐nine patients received NAAP, while 282 underwent AAP. In the unmatched comparison, NAAP patients had a lower BMI (25.1 ± 4.1 vs. 29.8 ± 4.3, *p <* 0.01) and were younger (57.4 ± 15.1 vs. 59.3 ± 16.5 years old, *p <* 0.041). The rate of smoking patients (18.6% vs. 9.6%, p = 0.001) and those with cardiovascular diseases (30.7% vs. 23.4%, p = 0.011) was higher in the NAAP cohort than in the AAP cohort. After PSM the EGD cohort included 282 patients with NAAP and 188 with AAP (Table 1).

**TABLE 1:** Baseline characteristics: esophagogastroduodenoscopies cohort.

	Unmatched (*n* = 2721)			Matched (*n* = 470)		
	NAAP (*n* = 2439)	AAP (*n* = 282)	*p‐*value	NAAP (*n* = 282)	AAP (*n* = 188)	*p‐*value
Age (years)	57.4±15.1	59.3±16.5	**0.04**	59.3±16.5	60.5±13.2	0.41
BMI (kg/m^2^)	25.1±4.1	29.8±4.3	**<0.001**	29.8±4.3	29.3±4.8	0.22
Sex^*^						
Male	1182 (48.5%)	156 (55.3%)	**0.03**	156 (55.3%)	98 (52.1%)	0.49
Female	1255 (51.5%)	126 (44.6%)		126 (44.7%)	90 (47.9%)	
Smoking^*^						
Yes	451 (18.6%)	27 (9.6%)	**<0.001**	27 (9.6%)	17 (9.0%)	0.84
No	1977 (81.4%)	255 (90.4%)		255 (90.4%)	171 (91%)	
ASA score^*^						
1	1218 (50.0%)	135 (47.9%)	0.49	135 (47.9%)	108 (57.4%)	0.05
2	1217 (50.0%)	147 (52.1%)		147 (52.1%)	80 (42.6%)	
Allergies^*^						
Yes	584 (24.1%)	72 (25.5%)	0.59	72 (25.5%)	39 (21.0%)	0.25
No	1841 (75.9%)	210 (74.5%)		210 (74.5%)	147 (79.0%)	
Diabetes^*^						
Yes	205 (8.4%)	21 (7.4%)	0.56	21 (7.4%)	21 (11.2%)	0.16
No	2224 (91.6%)	261 (92.6%)		261 (92.6%)	167 (88.8%)	
Bronchopulmonary diseases^*^						
Yes	168 (6.9%)	24 (8.5%)	0.33	24 (8.5%)	15 (8.0%)	0.85
No	2252 (93.1%)	258 (91.5%)		258 (91.5%)	172 (92.0%)	
Cardiovascular diseases^*^						
Yes	746 (30.7%)	66 (23.4%)	**0.01**	66 (23.4%)	51 (27.1%)	0.36
No	1685 (69.3%)	216 (76.6%)		216 (76.4%)	137 (72.9%)	

Abbreviations: AAP, anesthesiologist administration of propofol; ASA, American Society of Anesthesiologists; BMI, body mass index; NAAP, non‐anesthesiologist administration of propofol.

* Not available for every patient due to missing data.

#### 3.2.2 Colonoscopies Cohort

Two thousand four hundred ninety‐one patients received NAAP and 257 underwent AAP. Patients in the NAAP group were younger (60.2 ± 13.9 vs. 63.0 ± 16.0 years old, *p* = 0.002) and had a lower BMI (25.3 ± 3.8 vs. 29.1 ± 4.3, *p <* 0.001) compared to the AAP group. The NAAP group also included fewer smokers (18.0% vs. 12.0%, *p* = 0.017), fewer allergic patients (24.0% vs. 32.3%, *p* = 0.004), and fewer with diabetes (7.4% vs. 13.6%, *p <* 0.001). After PSM the colonoscopy cohort consisted of 256 patients with NAAP and 174 with AAP (Table 2)

**TABLE 2:** Baseline characteristics: colonoscopies cohort.

	Unmatched (*n* = 2748)			Matched (*n* = 430)		
	NAAP (*n* = 2491)	AAP (*n* = 257)	*p*‐value	NAAP (*n* = 256)	AAP (*n* = 174)	*p*‐value
Age (years)	60.2± 13.9	63.0± 16.0	**0.002**	63.0± 15.0	63.0± 13.2	0.93
BMI (kg/m^2^)	25.3± 3.8	29.1± 4.3	**<0.001**	29.1± 4.3	28.9± 4.1	0.63
Sex^*^						
Male	1153 (46.3%)	115 (44.7%)	0.62	114 (44.5%)	83 (47.7%)	0.51
Female	1336 (53.7%)	142 (55.3%)		142 (55.5%)	91 (52.3%)	
Smoking^*^						
Yes	447 (18.0%)	31 (12.0%)	0.01	31 (12.1%)	22 (12.6%)	0.86
No	2036 (82.0%)	226 (88.0%)		225 (87.9%)	152 (87.4%)	
ASA score^*^						
1	1234 (49.8%)	133 (51.8%)	0.54	124 (48.4%)	77 (44.3%)	0.39
2	1246 (50.2%)	124 (48.3%)		132 (51.6%)	97 (55.7%)	
Allergies^*^						
Yes	596 (24.0%)	83 (32.3%)	**0.004**	83 (32.4%)	52 (29.9%)	0.57
No	1881 (76.0%)	174 (67.7%)		173 (67.6%)	122 (70.1%)	
Diabetes^*^						
Yes	183 (7.4%)	35 (13.6%)	**<0.001**	35 (13.7%)	22 (12.6%)	0.75
No	2285 (92.6%)	222 (86.4%)		221 (86.3%)	152 (87.4%)	
Bronchopulmonary diseases^*^						
Yes	123 (5.0%)	13 (5.0%)	0.96	13 (5.1%)	14 (8.2%)	0.19
No	2338 (95.0%)	244 (95.0%)		243 (94.9%)	156 (91.8%)	
Cardiovascular diseases^*^						
Yes	773 (31.2%)	94 (36.6%)	0.08	93 (36.3%)	66 (37.9%)	0.73
No	1701 (68.8%)	163 (63.4%)		163 (63.7%)	108 (62.1%)	

Abbreviations: AAP, anesthesiologist administration of propofol; ASA, American Society of Anesthesiologists; BMI, body mass index; NAAP, non‐anesthesiologist administration of propofol.

* Not available for every patient due to missing data.

#### 3.2.3 Covariate Balance and Matching Performance

After PSM, all baseline covariates were adequately balanced between the NAAP and AAP groups in both the EGDs and colonoscopy cohorts, with all SMDs below the conventional threshold of 0.2 (Tables S5 and S6).

C‐statistics were 0.80 for the EGD cohort and 0.76 for the colonoscopy cohort, indicating the acceptable discriminatory ability of the propensity score models.

### 3.3 Outcomes

#### 3.3.1 EGDs Cohort

In the NAAP group, 24 AEs (1.1%) were reported, including 11 cases of hypoxia (0.5%), three of bradycardia (0.1%), nine of hypotension (0.4%), and one case involving two or more AEs (0.1%). All AEs were managed by the endoscopists without major consequences. In the AAP group, one case of hypoxia (0.4%) and one of bradycardia (0.4%) were recorded. Post‐PSM comparisons revealed no significant AEs rate difference between the two cohorts (NAAP 0.4% vs. AAP 1.0%, *p* = 0.452).

In the unmatched cohorts, the procedure time was longer for the NAAP group compared to the AAP group (17.3 ± 6.0 vs. 16.4 ± 6.5 min, *p <* 0.001).

Even post‐matching, propofol dosage was higher for patients in the AAP group compared to those in the NAAP group (288.2 ± 121.8 mg vs. 136.4 ± 49.4 mg, *p <* 0.001; Table 3).

**TABLE 3:** Adverse events: esophagogastroduodenoscopies cohort.

	Unmatched (*n* = 2721)			Matched (*n* = 470)		
	NAAP (*n* = 2439)	AAP (*n* = 282)	*p‐*value	NAAP (*n* = 282)	AAP (*n* = 188)	*p‐*value
Adverse events			0.81^*^			0.45^**^
None Hypoxia Bradycardia Hypotension >2 events	2415 (98.9%) 11 (0.5%) 3 (0.1%) 9 (0.4%) 1 (0.1%)	281 (99.2%) 1 (0.4%) 1 (0.4%) 0 (0%) 0 (0%)		281 (99.6%) 1 (0.4%) 0 (0%) 0 (0%) 0 (0%)	186 (99.0%) 1 (0.5%) 1 (0.5%) 0 (0%) 0 (0%)	
Procedure duration (minutes)	17.3± 6.0	16.4± 6.5	**0.03**	16.4± 6.5	17.0± 5.4	0.32
Propofol dosage (mg)	126.76± 44.4	288.2± 121.8	**<0.001**	136.4± 49.4	288.2± 121.8	**<0.001**

Abbreviations: AAP, anesthesiologist administration of propofol; NAAP, non‐anesthesiologist administration of propofol.

* Fisher's exact test: 1.0.

** Fisher's exact test: 0.57.

#### 3.3.2 Colonoscopies Cohort

Forty‐four patients (1.7%) of the NAAP cohort experienced AEs, including 25 cases of hypoxia (1%), 11 of bradycardia (0.4%), three of hypotension (0.1%), and five involving two or more AEs (0.2%). Like the EGDs cohort, these events were effectively managed by the endoscopist and did not result in severe outcomes. In the AAP group, there were six cases of hypoxia (2.3%), two of bradycardia (0.8%), and one of hypotension (0.4%). Post‐PSM comparisons showed no difference in the AEs rate between the two cohorts (NAAP 3.5% vs. AAP 0.6%, *p* = 0.249).

No difference in procedure time was found between the two cohorts (overall NAAP 31.6 ± 13.7 vs. 30.8 ± 15.9 min, *p* = 0.37).

Propofol dosage was lower in the NAAP group (after PSM, 141.19 ± 65.1 mg vs. 424.1 ± 254.4 mg, *p <* 0.001; Table 4).

**TABLE 4:** Adverse events: colonoscopies cohort.

	Unmatched (*n* = 2748)			Matched (*n* = 430)		
	NAAP (*n* = 2491)	AAP (*n* = 257)	*p‐*value	NAAP (*n* = 256)	AAP (*n* = 174)	*p‐*value
Adverse events			*0.20* ^*^			*0.24* ^**^
None	2447 (98.2%)	248 (96.5%)		247 (96.5%)	173 (99.4%)	
Hypoxia	25 (1.0%)	6 (2.3%)		6 (2.3%)	1 (0.6%)	
Bradycardia	11 (0.4%)	2 (0.8%)		2 (0.8%)	0 (0%)	
Hypotension	3 (0.1 %)	1 (0.4%)		1 (0.4%)	0 (0%)	
>2 events	5 (0.2%)	0 (0%)		0 (0%)	0 (0%)	
Procedure duration (minutes)	31.6± 13.7	30.8± 15.9	*0.37*	30.8± 16.0	31± 14.0	*0.88*
Propofol dosage (mg)	148.8± 69.6	425.4± 254.6	***<0.001***	141.19± 65.1	424.1 ± 254.4	***<0.001***

Abbreviations: AAP, anesthesiologist administration of propofol; NAAP, non‐anesthesiologist administration of propofol.

* Fisher's exact test: 0.09.

** Fisher's exact test: 0.06.

### 3.4 Budget Impact Model

In 2023, 4227 EGDs and 4327 colonoscopies were performed at IRCCS San Raffaele Hospital.

A total of 48,573 EGDs and 37,530 colonoscopies were conducted across the population served by ATS Milan and 127,716 EGDs and 93,260 colonoscopies in Lombardy in 2023. Nationally, it was estimated that 755,100 EGDs and 551,384 colonoscopies were performed.

In Lombardy, it was estimated that 128,011 EGDs and 93,475 colonoscopies would be performed in 2024, and 103,110 EGDs and 75,292 colonoscopies in 2025. Across Italy, projections suggest that 754,371 EGDs overall (625,876 in low‐risk patients) and 550,852 colonoscopies (503,622 in low‐risk patients) would be carried out in 2024, and 752,960 EGDs overall (624,706 in low‐risk patients) and 549,822 colonoscopies (502,680 in low‐risk patients) in 2025 (Table 5).

**TABLE 5:** Overall population and low‐risk patients’ population undergoing endoscopy at IRCSS San Raffaele Hospital, ATS of Milan, Lombardy, and Italy in the 2023–2025 triennium

	2023	2024	2025
**EGDs**			
*IRCCS San Raffaele Hospital*			
Overall population	4227	4237	4245
Low‐risk patients	3507	3515	3522
*ATS Milan*			
Overall population	48,573	48,685	48,774
Low‐risk patients	40,299	40,392	40,466
*Lombardy*			
Overall population	127,716	128,011	103,110
Low‐risk patients	105,962	106,206	85,547
*Italy*			
Overall population	755,100	754,371	752,960
Low‐risk patients	626,481	625,876	624,706
**Colonoscopies**			
*IRCCS San Raffaele Hospital*			
Overall population	4327	4337	4345
Low‐risk patients	3956	3965	3972
*ATS Milan*			
Overall population	37,530	37,617	37,686
Low‐risk patients	34,312	34,392	34,455
*Lombardy*			
Overall population	93,260	93,475	75,292
Low‐risk patients	85,264	85,460	68,837
*Italy*			
Overall population	551,384	550,852	549,822
Low‐risk patients	504,108	503,622	502,680

#### 3.4.1 Budget Impact Analysis

Table 6 shows the budget impact results on overall costs for low‐risk patients undergoing endoscopy in the 2023–2025 period.

**TABLE 6:** Budget impact results on overall costs (€) for low‐risk patients undergoing endoscopy in the 2023–2025 triennium.

	IRCCS San Raffaele Hospital (€)					ATS Milan (€)				Lombardy (€)				Italian National Health System (€)			
	Single procedure	2023	2024	2025	2023–2025 triennium	2023	2024	2025	2023–2025 triennium	2023	2024	2025	2023–2025 triennium	2023	2024	2025	2023–2025 triennium
**AAP (SC1)**	**196**	**738,728**	**740,451**	**741,829**	**2,221,008**	**7,205,160**	**7,221,822**	**7,235,049**	**21,662,031**	**18,374,328**	**18,416,703**	**14,834,251**	**51,625,282**	**108,635,142**	**108,530,301**	**108,327,325**	**325,492,767**
EGD	8074	283,155	283,825	284,361	851,341	3,253,773	3,261,275	3,267,237	9,782,285	8,555,346	8,575,076	6,907,036	24,037,459	50,582,053	50,533,237	50,438,729	151,554,019
Colonoscopy	11,516	455,573	456,626	457,468	1,369,667	3,951,387	3,960,547	3,967,812	11,879,747	9,818,982	9,841,627	7,927,215	27,587,823	58,053,089	57,997,064	57,888,596	173,938,749
**NAAP (SC2)**	**121**	**455,462**	**456,524**	**457,374**	**1,369,359**	**4,443,910**	**4,454,187**	**4,462,345**	**13,360,443**	**11,333,521**	**11,359,658**	**9,149,956**	**31,843,135**	**67,007,547**	**66,942,880**	**66,817,681**	**200,768,108**
EGD	4994	175,140	175,554	175,885	526,579	2,012,551	2,017,192	2,020,880	6,050,623	5,291,726	5,303,930	4,272,200	14,867,856	31,286,447	31,256,253	31,197,797	93,740,497
Colonoscopy	7086	280,322	280,970	281,488	842,780	2,431,359	2,436,995	2,441,465	7,309,820	6,041,794	6,055,728	4,877,757	16,975,279	35,721,100	35,686,627	35,619,885	107,027,611
**Savings**	**75**	**283,266**	**283,927**	**284,455**	**851,649**	**2,761,249**	**2,767,635**	**2,772,704**	**8,301,589**	**7,040,807**	**7,057,045**	**5,684,295**	**19,782,147**	**41,627,595**	**41,587,421**	**41,509,643**	**124,724,659**
EGD	3080	108,016	108,271	108,476	324,762	1,241,221	1,244,083	1,246,357	3,731,662	3,263,620	3,271,146	2,634,837	9,169,603	19,295,606	19,276,984	19,240,932	57,813,522
Colonoscopy	4430	175,251	175,656	175,980	526,886	1,520,028	1,523,552	1,526,347	4,569,927	3,777,187	3,785,898	3,049,458	10,612,544	22,331,989	22,310,437	22,268,711	66,911,137

Abbreviations: AAP (SC1), anesthesiologist administration of propofol (scenario 1); EGD, esophagogastroduodenoscopy; NAAP (SC2), non‐anesthesiologist administration of propofol (scenario 2).

*Notes*: **Anesthesiologist Administration of Propofol (scenario 1)**: Low‐risk patients undergoing deep sedation managed by an anesthesiologist. **Non‐Anesthesiologist Administration of Propofol (scenario 2)**: Low‐risk patients undergoing moderate sedation managed by the endoscopist, without the direct involvement of the anesthesiologist. **Savings**: Reduction in € resulting from the adoption of scenario 2 instead of scenario 1 at IRCCS San Raffaele Hospital, ATS Milan, Lombardy Region, and the Italian National Health System (NHS).

Over the 2023–2025 triennium, the use of NAAP instead of AAP would result in savings of €851,649 at San Raffaele Hospital, €8,301,589 at the ATS of Milan, and €19,782,147 in Lombardy. Nationally, the implementation of NAAP instead of AAP was projected to lead to cumulative savings of €124,724,659.

For the Italian NHS, the use of NAAP instead of AAP would also result in savings of €28,510,374 in drug costs and a reduction in staff workload equivalent to 2223 working days over the 2023–2025 period (Tables S7 and S8).

#### 3.4.2 Sensitivity Analysis

**FIGURE 2:**
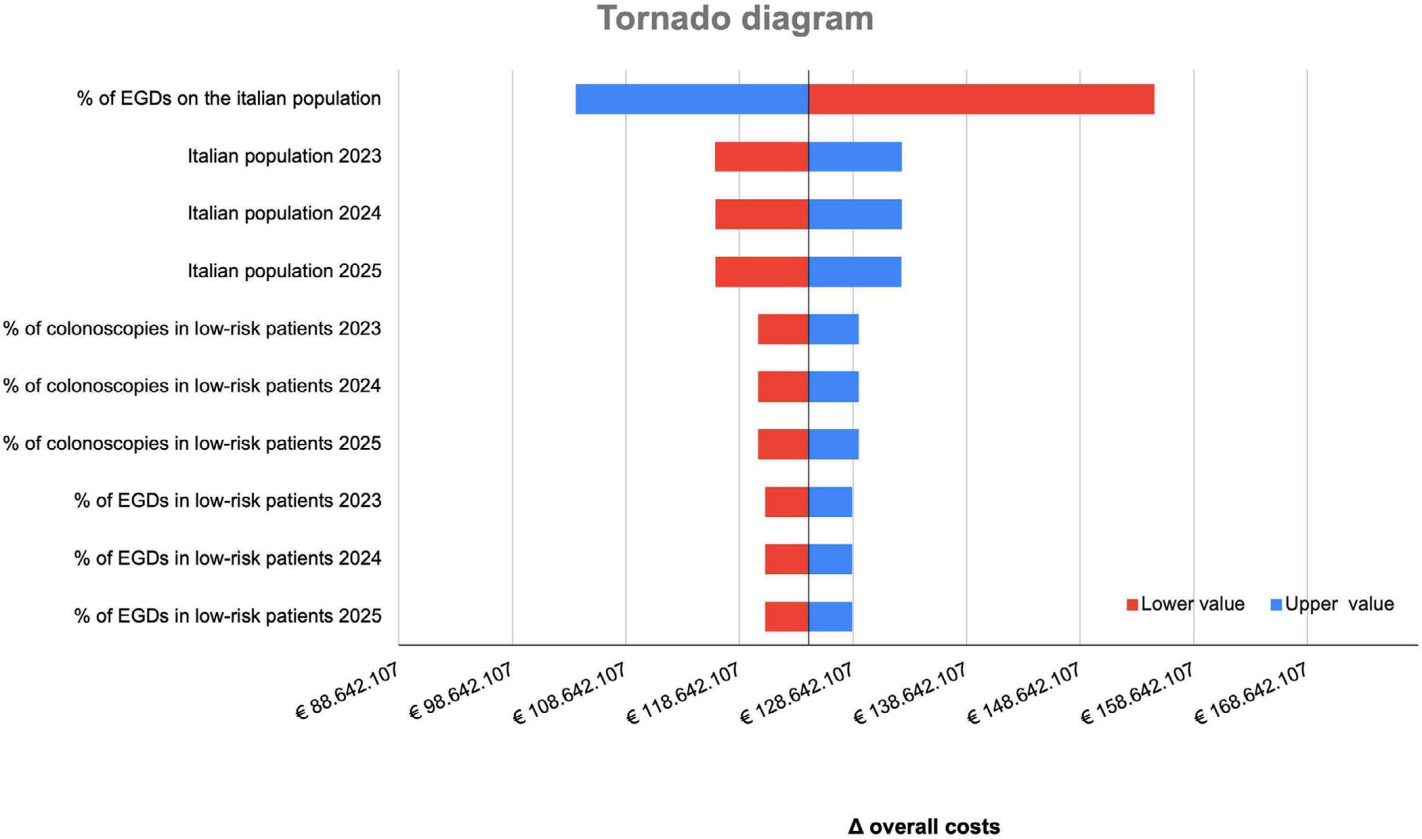
Tornado diagram from the sensitivity analysis. It ranks input variables by their impact on the overall budget difference between non‐anesthesiologist‐administered propofol and anesthesiologist‐administered‐propofol. The rate of the population undergoing EGD had the greatest effect: a ±10% variation shifted the cost impact between €8.0 and €11.9 million. Shorter bars indicate parameters with lower influence. Red and blue bars represent lower and upper input values, respectively. EGD: esophagogastroduodenoscopy.

The sensitivity analysis of overall cost is presented in Figure 2. As shown, the BIM was most sensitive to the rate of the population undergoing EGD: a variation of ± 10% would result in the incremental budget impact ranging from €8,002,928.36 to €11,904,853.64.

## 4 Discussion

In this study, we found that the AE rate with NAAP was comparable to AAP for low‐anesthesiologic‐risk patients undergoing outpatient endoscopy. Furthermore, we demonstrated that implementing NAAP instead of AAP in a low‐risk population (ASA 1 and 2) could lead to savings of approximately €124 million and free up to 2223 working days for healthcare professionals over the 2023–2025 period for the Italian NHS.

Over the past 15 years, propofol‐based sedation has become the preferred option for many endoscopists in outpatient endoscopy. Numerous studies have shown that propofol sedation results in higher satisfaction rates among patients and endoscopists, as well as shorter sedation and recovery times compared to the combined use of opioids and benzodiazepines [16, 17, 18].

However, the adoption of NAAP remains limited in many Western countries. In the US, the use of AAP for outpatient endoscopic procedures in low‐risk patients increased from 14% in 2003 to 48% in 2013 for Medicare patients, and from 14% to 53% among privately insured patients [19, 20, 21]. Although more recent data is limited, this trend appears to have continued. Currently, NAAP is routinely practiced in only a minority of European countries.

The use of AAP for outpatient endoscopy is not supported by improved outcomes. First, AAP is not associated with a higher adenoma detection rate during colonoscopy [22]. Secondly, AAP may not be safer than NAAP. Available data show either similar complication rates [23, 24] or higher rates with AAP compared to NAAP [25, 26, 27]

A major obstacle to broader NAAP adoption in Western countries is the regulatory restriction that limits propofol administration to anesthesiologists. While European and American gastrointestinal guidelines endorse NAAP for low‐risk procedures, provided proper training is ensured [7, 28], anesthesiology societies argue that NAAP is unsafe and that propofol should only be administered by those trained in general anesthesia [29].

In our study, AE rates were similar between NAAP and AAP in both unmatched and matched cohorts. In the overall NAAP cohort, the AE rate was 1.2% (68/5469), with hypoxia being the most common (0.6%). All AEs were minor and managed by the endoscopist without anesthesiologist intervention.

Large population‐based studies support the safety of NAAP when managed by trained personnel. In the ProSed study (368,206 procedures across 39 German centers), minor complications occurred in 0.3%, and major complications in only 0.01% [5]. Similarly, Rex et al. reported only 11 cases of intubation in 646,080 NAAP procedures (6).

Our findings are consistent with the meta‐analysis by Daza et al. [30], which included 21,054 ASA I–II patients across five studies comparing NAAP and AAP. That analysis found no increase in airway intervention or hypotension with NAAP. Although higher bradycardia rates were reported, the two included randomized control trials [31, 32] showed no statistically significant differences between groups.

In our cohort, the mean propofol dose in the AAP group was roughly twice as high for EGDs and three times as high for colonoscopies compared to NAAP. This likely reflects different sedation depth thresholds. Despite this, AE rates remained low in both groups, suggesting that higher doses did not translate into increased risk.

Few studies have assessed the economic cost of AAP in low‐risk patients. Most cost‐effectiveness analyses focus on colorectal cancer (CRC) screening. In the United States and France, anesthesiologist involvement was estimated to increase screening costs by 20% and 285%, respectively [19, 33]. Hassan et al. projected that using NAAP in CRC screening could save $3.2 billion in the US and €0.8 billion in France over 10 years [34].

Our analysis found that replacing AAP with NAAP in low‐risk patients could save over €124 million and 2,223 working days over three years for the Italian NHS. Broader NAAP adoption could generate substantial cost savings and better allocate healthcare resources, allowing anesthesia professionals to focus on higher‐risk cases.

In our sensitivity analysis, the EGD rate procedures were the most influential parameter. Inappropriate use of EGDs procedures is a known issue, with rates as high as 77% [35]. Ensuring appropriate indications will be crucial to maximizing NAAP‐related savings.

It is possible that our analysis either underestimated or overestimated the economic benefits of NAAP in the Italian NHS. Underestimation may have occurred because our model did not account for colonoscopies performed as part of CRC screening, which could increase due to an aging population and evolving guidelines recommending earlier screening.

Conversely, we may have overestimated the benefits, as our analysis did not include the costs of training nurses and medical staff, which may represent a relevant budget component in the real‐world implementation of NAAP programs. Hassan's study estimated $47 million to train 17,166 US nurses [34], suggesting that similar costs may apply to the Italian NHS. It is important to note that physician training is essential to ensure the safe administration of propofol [7]. Additionally, local healthcare policies were not included in the model, as they vary widely across regions and were beyond the study's scope.

The AE rate observed in our study should also be interpreted with caution. Given the low frequency of severe complications related to propofol‐based sedation [5, 6], the overall sample size limits the ability to detect major AEs. Furthermore, the reduced sample size in the propensity score‐matched cohort may not be sufficient to fully reflect the actual rate of AEs. Another limitation is that patient satisfaction was not assessed, although it may influence both acceptance and long‐term adherence to NAAP programs.

In conclusion, our study confirms that NAAP via TCI has a comparable AEs rate to AAP in low‐risk patients, based on a cohort of over 5000 individuals treated at a single tertiary center. Broader adoption of NAAP could result in substantial cost savings for the Italian NHS, exceeding €100 million and 2,000 working days between 2023 and 2025.

In the future, revising recommendations to clarify indications, training, and safety standards for NAAP will be essential. Equally important is fostering collaboration between anesthesiologists and endoscopists through shared protocols, joint training, and institutional support. This integrated approach would promote the safe implementation of NAAP and reduce unnecessary healthcare costs.

## Ethics Statement

Approval of the research protocol by an Institutional Reviewer Board: Protocol REG_EGDS COLON, approval Code: 79/12/2022).

## Consent

All patients provided informed consent for the use of their data for scientific purposes.

## Conflicts of Interest

The authors declare no conflicts of interest.

## Supporting information




**Supporting Table 1**: Exclusion criteria.
**Supporting Table 2**: Model input on overall costs.
**Supporting Table 3**: Healthcare professional workload based on single procedures.
**Supporting Table 4**: Drugs costs.
**Supporting Table 5**: Standardized mean differences for baseline covariates before and after propensity score matching in the EGDs cohort.
**Supporting Table 6**: Standardized mean differences for baseline covariates before and after propensity score matching in the colonoscopies cohort.
**Supporting Table 7**: Budget impact results on drugs costs (€) for low‐risk patients undergoing endoscopy in the 2023–2025 triennium.
**Supporting Table 8**: Budget impact results on staff's time involvement (days) for low‐risk patients undergoing endoscopy in the 2023–2025 triennium.
