# Supplementary material for: Impact of Non‐Anesthesiologist‐Administered Propofol Sedation for Outpatient Endoscopy in the Healthcare System
Source: DEN Open. 2025 Jun 5;6(1):e70151. doi: 10.1002/deo2.70151 (PMC12140971; doi:10.1002/deo2.70151)
Supplement: Supplementary file 1 — Supporting Table 1: Exclusion criteria. Supporting Table 2: Model input on overall costs. Supporting Table 3: Healthcare professional workload based on single procedures. Supporting Table 4: Drugs costs. Supporting Table 5: Standardized mean differences for baseline covariates before and after propensity score matching in the EGDs cohort. Supporting Table 6: Standardized mean differences for baseline covariates before and after propensity score matching in the colonoscopies cohort. Supporting Table 7: Budget impact results on drugs costs (€) for low‐risk patients undergoing endoscopy in the 2023–2025 triennium. Supporting Table 8: Budget impact results on staff's time involvement (days) for low‐risk patients undergoing endoscopy in the 2023–2025 triennium. [file DEO2-6-e70151-s001.docx]

**Supplementary material**

**1s. Sedation modalities**

*Non-Anesthesiologist Administration of Propofol (NAAP)*

Sedation reached a “moderate sedation” level, defined as the patient’s ability to respond to verbal or tactile stimulation while maintaining an open airway and stable cardiovascular function [1]. A specialized nurse managed the sedation under the endoscopist's supervision. An on-call anesthesiology was always available in the department.

The propofol concentration was set at 1.2–1.6 g/ml, adjusted in increments of 0.1 g/ml up to 2 g/ml if needed. For colonoscopy, intravenous fentanyl (1 µg/kg) was given additionally. Monitoring included continuous SpO2 and heart rate assessment, and non-invasive blood pressure every 5 minutes. Oxygen (2 L/min) was administered via nasal cannula and adjusted as needed. After the procedure, patients were moved to a recovery area and evaluated every 5 minutes until fit for discharge, determined by a Modified Aldrete Score of 9 or more [2].

*Anesthesiologist Administration of Propofol (AAP).*

Sedation reached a "deep sedation" level, where patients were not easily aroused but could respond to painful stimuli, while maintaining spontaneous breathing and stable cardiovascular function [1]. Sedation was managed by senior anesthesiologists or fellows under supervision, assisted by a nurse.

Initial propofol doses were 4-5 mg/kg for patients under 80 and 2-3 mg/kg for those aged 80 or above. Deep sedation was confirmed by a score of 1 on the Observer’s Assessment of Alertness/Sedation Scale [3]. Fentanyl was administered at the anesthesiologist’s discretion.

Monitoring included electrocardiography, SpO2, respiratory rate, and blood pressure every 5 minutes. All patients received oxygen (minimum 4 L/min). After the procedure, patients were moved to recovery while still asleep and continuously monitored until they awoke. Discharge criteria were the same as for NAAP.

**References**

1. ASGE Standards of Practice Committee, Early DS, Lightdale JR, et al. Guidelines for sedation and anesthesia in GI endoscopy. Gastrointest Endosc. 2018; 87: 327-37.
2. Aldrete JA, Kroulik D. A postanesthetic recovery score. Anesth Analg. 1970; 49: 924-34.
3. Chernik DA, Gillings D, Laine H, et al. Validity and reliability of the Observer's Assessment of Alertness/Sedation Scale: study with intravenous midazolam. J Clin Psychopharmacol. 1990; 10: 244-51.

**2s. Adverse events definition**

- Hypotension: systolic blood pressure <90 mmHg or a decrease > 50 mmHg from baseline.
- Hypoxia: SpO2 <90% for over 10 seconds.
- Agitation: increased restlessness after propofol administration.
- Bradycardia: heart rate < 40 bpm.

**3s. Propensity score matching**

Propensity score matching (PSM) was adopted to balance baseline differences between the NAAP and AAP groups, based on age, sex, body mass index (BMI), smoking status, ASA score, and relevant comorbidities (diabetes, cardiovascular and bronchopulmonary disease).

Logistic regression models were used to estimate propensity scores, followed by nearest-neighbor matching without replacement, applying a caliper width of 0.1 standard deviations of the logit of the propensity score. This threshold was chosen to reduce residual bias while maintaining an adequate number of matched pairs, in line with established recommendations [1]

The distribution of propensity scores was examined before and after matching to confirm adequate overlap (common support). Participants falling outside the common support region were excluded to ensure valid comparisons and avoid extrapolation.

The effectiveness of the matching procedure was assessed by calculating standardized mean differences (SMDs) for baseline covariates before and after matching. SMD <0.2 was considered indicative of acceptable balance [2].

The discriminatory capacity of the propensity score model was assessed using the C-statistic (area under the ROC curve), where values of 0.5 indicate no discrimination, 0.7–0.8 acceptable discrimination, and values above 0.8 excellent discrimination [3].

**References**

1. Austin PC. An Introduction to Propensity Score Methods for Reducing the Effects of Confounding in Observational Studies. Multivariate Behav Res. 2011; 46: 399-424.
2. Stuart EA. Matching methods for causal inference: A review and a look forward. Stat Sci. 2010 Feb 1; 25: 1-21.
3. Hosmer DW, Lemeshow S. Applied logistic regression. 2nd ed. New York: Wiley; 2000.

**Supplementary Table 1. Exclusion criteria**

| Emergency procedures |
| --- |
| ASA ≥3 |
| Mallampati class > 3 |
| Obstructive Sleep Apna Syndrome |
| BMI >40 |
| Hemodynamic instability |
| Pregnant women |
| Severe COPD |
| Poorly cooperative patients (psychiatric or dementia patients, cerebrovascular patients, alcoholics in a state of psychomotor agitation) |
| Allergy to the drugs used or its components |
| Combined procedures (Esophagogastroduodenoscopy + Colonoscopy) |
| Interventional procedures (Esophagogastroduodenoscopy +/- biopsy > 45 minutes, Colonoscopy +/- biopsy > 60 minutes, Esophagogastroduodenoscopy + polypectomy/Endoscopic Mucosal Resection, Colonoscopy + polypectomy/Endoscopic Mucosal Resection, Esophagogastroduodenoscopy + nasojejunal tube placement, Esophagogastroduodenoscopy + botulinum toxin injection, Esophagogastroduodenoscopy + Radiofrequency for Barretts esophagus treatment |
| Rectosigmoidoscopies |

ASA American Society of Anaesthesiologists, BMI Body Mass Index, COPD Chronic Obstructive Pulmonary Disease

**Supplementary Table 2. Model input on overall costs**

| **EGDs** |  |  |  |  |
| --- | --- | --- | --- | --- |
|  | **Preparation** | **Procedure** | **Monitoring** | **Total** |
| NAAP | 9.26 € | 40.11 € | 0.57 € | **49.94 €** |
| AAP | 9.26 € | 67.11 € | 4.37 € | **80.74 €** |
| **Colonoscopy** |  |  |  |  |
|  | **Preparation** | **Procedure** | **Monitoring** | **Total** |
| NAAP | 9.26 € | 61.03 € | 0.57 € | **70.86** € |
| AAP | 9.26 € | 101.53 € | 4.37 € | **115.16** € |

EGDs Esophagogastroduodenoscopies, NAAP Non-Anaesthesiologist Administration of Propofol, AAP Anaesthesiologist Administration of Propofol

*Notes: Costs were obtained from the San Raffaele Hospital Health Management Department*

**Supplementary Table 3. Healthcare professional workload based on single procedures**

| **EGD** |  |  |  |  |
| --- | --- | --- | --- | --- |
|  | **Preparation**** | **Procedure** | **Monitoring** | **Total***** |
| *NAAP (min)* |  |  |  |  |
| Anaesthetist | 0 | 0 | 0 | **0** |
| Endoscopist | 0 | 30 | 0 | **30** |
| Nurses (two) | 5x2=10 | 30x2=60 | 0 | **70** |
| *AAP (min)* |  |  |  |  |
| Anaesthetist | 5 | 30 | 5 | **40** |
| Endoscopist | 0 | 30 | 0 | **30** |
| Nurses (two) | 5x2=10 | 30x2=60 | 20* | **90** |
| **Colonoscopy** |  |  |  |  |
|  | **Preparation**** | **Procedure** | **Monitoring** | **Total** |
| *NAAP (min)* |  |  |  |  |
| Anaesthetist | 0 | 0 | 0 | **0** |
| Endoscopist | 0 | 45 | 0 | **45** |
| Nurses (two) | 5x2=10 | 45x2=90 | 0 | **100** |
| *AAP (min)* |  |  |  |  |
| Anaesthetist | 5 | 45 | 0 | **50** |
| Endoscopist | 0 | 45 | 0 | **45** |
| Nurses (two) | 5x2=10 | 45x2=90 | 20* | **120** |

*1 nurse every 6 patients

** *Reception staff (patients check-in) and Healthcare Assistant costs not included*

****Recovery room space not included*

**Supplementary Table 4. Drugs costs**

| **EGDs*** |  |  |  |
| --- | --- | --- | --- |
|  | **Dosage (mg)** | **Cost for vial, 50 mg** | **Cost per procedure (€)** |
| *NAAP* |  |  |  |
| Propofol | 136 | 2€ | **5€** |
| *AAP* |  |  |  |
| Propofol | 288 | 2€ | **12€** |
| **Colonoscopies*** |  |  |  |
|  | **Dosage (mg)** | **Cost for vial, 50 mg** | **Cost per procedure (€)** |
| *NAAP* |  |  |  |
| Propofol | 141 | 2€ | **6€** |
| *AAP* |  |  |  |
| Propofol | 424 | 2€ | **17€** |

******Fentanyl used for sedation and drugs/devices used for the management of minor adverse events (i,e, atropine for bradycardia and goggles for hypoxia), were not included in the analysis due to their negligible costs.*

**Supplementary Table 5. Standardized mean differences for baseline covariates before and after propensity score matching in the EGDs cohort.**

| **Variable** | **SMD before matching** | **SMD after matching** |
| --- | --- | --- |
| Age (years) | 0.12 | 0.08 |
| BMI (kg/m²) | 1.12 | 0.11 |
| Sex (Male) | 0.14 | 0.064 |
| Smoking (Yes) | 0.26 | 0.018 |
| ASA score = 1 | 0.041 | 0.193 |
| Allergies (Yes) | 0.0 | 0.056 |
| Diabetes (Yes) | 0.0 | 0.033 |
| Bronchopulmonary diseases (Yes) | 0.0 | 0.119 |
| Cardiovascular diseases (Yes) | 0.14 | 0.034 |

*SMD Standardized mean differences*

All SMDs after matching were <0.2, confirming adequate balance between groups.

**Supplementary Table 6. Standardized mean differences for baseline covariates before and after propensity score matching in the colonoscopies cohort.**

| **Variable** | **SMD before matching** | **SMD after matching** |
| --- | --- | --- |
| Age (years) | 0.12 | 0.0 |
| BMI (kg/m²) | 1.12 | 0.048 |
| Sex (Male) | 0.14 | 0.064 |
| Smoking (Yes) | 0.26 | 0.016 |
| ASA score = 1 | 0.041 | 0.084 |
| Allergies (Yes) | 0.0 | 0.057 |
| Diabetes (Yes) | 0.0 | 0.033 |
| Bronchopulmonary diseases (Yes) | 0.0 | 0.119 |
| Cardiovascular diseases (Yes) | 0.14 | 0.034 |

*SMD Standardized mean differences*

All SMDs after matching were <0.2, confirming adequate balance between groups.

**Supplementary Table 7. Budget impact results on drugs costs (€) for low-risk patients undergoing endoscopy in the 2023-2025 triennium**

|  | IRCCS San Raffaele Hospital (€) | | | | ATS Milan (€) | | | | Lombardy (€) | | | | Italian National Health System (€) | | | |
| --- | --- | --- | --- | --- | --- | --- | --- | --- | --- | --- | --- | --- | --- | --- | --- | --- |
|  | 2023 | 2024 | 2025 | 2023-2025 triennium | 2023 | 2024 | 2025 | 2023-2025 triennium | 2023 | 2024 | 2025 | 2023-2025 triennium | 2023 | 2024 | 2025 | 2023-2025 triennium |
| **AAP (SC1)** | **107,494** | **107,745** | **107,946** | **323,185** | **1,046,183** | **1,048,603** | **1,050,523** | **3,145,309** | **2,666,753** | **2,672,903** | **2,677,817** | **8,017,473** | **15,766,731** | **15,751,515** | **15,722,056** | **47,240,303** |
| EGD | 40,401 | 40,496 | 40,573 | 121,470 | 464,249 | 465,319 | 466,170 | 1,395,738 | 1,220,679 | 1,223,494 | 1,225,743 | 3,669,915 | 7,217,058 | 7,210,093 | 7,196,608 | 21,623,759 |
| Colonoscopy | 67,094 | 67,249 | 67,373 | 201,715 | 581,934 | 583,283 | 584,353 | 1,749,570 | 1,446,074 | 1,449,409 | 1,452,074 | 4,347,558 | 8,549,673 | 8,541,422 | 8,525,448 | 25,616,544 |
| **NAAP (SC2)** | **41,390** | **41,487** | **41,564** | **124,441** | **412,749** | **413,703** | **414,461** | **1,240,913** | **1,057,319** | **,059,758** | **1,061,706** | **3,178,784** | **6,251,225** | **6,245,192** | **6,233,512** | **18,729,929** |
| EGD | 19,078 | 19,123 | 19,159 | 57,361 | 219,229 | 219,734 | 220,136 | 659,099 | 576,432 | 577,761 | 578,823 | 1,733,016 | 3,408,055 | 3,404,766 | 3,398,398 | 10,211,220 |
| Colonoscopy | 22,312 | 22,363 | 22,405 | 67,080 | 193,521 | 193,969 | 194,325 | 581,815 | 480,888 | 481,997 | 482,883 | 1,445,768 | 2,843,170 | 2,840,426 | 2,835,114 | 8,518,709 |
| **Savings** | **66,104** | **66,258** | **66,382** | **198,744** | **633,434** | **634,899** | **636,062** | **1,904,395** | **1,609,433** | **1,613,145** | **1,616,111** | **4,838,690** | **9,515,506** | **9,506,323** | **9,488,544** | **28,510,374** |
| EGD | 21,323 | 21,373 | 21,413 | 64,109 | 245,020 | 245,585 | 246,034 | 736,640 | 644,247 | 645,733 | 646,920 | 1,936,900 | 3,809,003 | 3,805,327 | 3,798,210 | 11,412,539 |
| Colonoscopy | 44,782 | 44,885 | 44,968 | 134,636 | 388,414 | 389,314 | 390,028 | 1,167,756 | 965,186 | 967,412 | 969,191 | 2,901,790 | 5,706,504 | 5,700,997 | 5,690,334 | 17,097,835 |

AAP (SC1) Anaesthesiologist Administration of Propofol (scenario 1), NAAP (SC2) Non-Anaesthesiologist Administration of Propofol (scenario 2), EGD esophagogastroduodenoscopy

*Notes*

***Anaesthesiologist Administration of Propofol (scenario 1):*** *Low-risk patients undergoing deep sedation managed by an anaesthesiologist.*

***Non-Anaesthesiologist Administration of Propofol (scenario 2):*** *Low-risk patients undergoing moderate sedation managed by the endoscopist, without the involvement of the anaesthesiologist.*

***Savings:*** *Reduction in € resulting from the adoption of scenario 2 instead of scenario 1 at IRCCS San Raffaele Scientific Institute, ATS Milan, Lombardy Region, and the Italian National Health System (NHS)*

**Supplementary Table 8. Budget impact results** **on staff’s time involvement (days) for low-risk patients undergoing endoscopy in the 2023-2025 triennium**

|  | IRCCS San Raffaele Hospital (days) | | | | ATS Milan (days) | | | | Lombardy (days) | | | | Italian National Health System (days) | | | |
| --- | --- | --- | --- | --- | --- | --- | --- | --- | --- | --- | --- | --- | --- | --- | --- | --- |
|  | 2023 | 2024 | 2025 | 2023-2025 triennium | 2023 | 2024 | 2025 | 2023-2025 triennium | 2023 | 2024 | 2025 | 2023-2025 triennium | 2023 | 2024 | 2025 | 2023-2025 triennium |
| **AAP (SC1)** | **15** | **15** | **15** | **46** | **149** | **150** | **150** | **449** | **381** | **382** | **383** | **1146** | **2,255** | **2,252** | **2,248** | **6,755** |
| EGD | 6 | 6 | 6 | 18 | 70 | 70 | 70 | 210 | 184 | 184 | 185 | 553 | 1,088 | 1,087 | 1,085 | 3,259 |
| Colonoscopy | 9 | 9 | 9 | 28 | 79 | 80 | 80 | 239 | 197 | 198 | 198 | 593 | 1,167 | 1,166 | 1,164 | 3,496 |
| **NAAP (SC2)** | **10** | **10** | **10** | **31** | **100** | **100** | **101** | **301** | **256** | **256** | **257** | **769** | **1,513** | **1,511** | **1,508** | **4,533** |
| EGD | 4 | 4 | 4 | 12 | 47 | 47 | 47 | 140 | 123 | 123 | 123 | 369 | 725 | 724 | 723 | 2,173 |
| Colonoscopy | 6 | 6 | 6 | 19 | 54 | 54 | 54 | 161 | 133 | 134 | 134 | 401 | 788 | 787 | 785 | 2,360 |
| **Savings** | **5** | **5** | **5** | **15** | **49** | **49** | **49** | **148** | **125** | **126** | **126** | **377** | **742** | **741** | **740** | **2,223** |
| EGD | 2 | 2 | 2 | 6 | 23 | 23 | 23 | 70 | 61 | 61 | 62 | 184 | 363 | 362 | 362 | 1,086 |
| Colonoscopy | 3 | 3 | 3 | 9 | 26 | 26 | 26 | 78 | 64 | 64 | 64 | 193 | 379 | 379 | 378 | 1,136 |

AAP (SC1) Anaesthesiologist Administration of Propofol (scenario 1), NAAP (SC2) Non-Anaesthesiologist Administration of Propofol (scenario 2), EGD esophagogastroduodenoscopy

*Notes*

***Anaesthesiologist Administration of Propofol (scenario 1):*** *Low-risk patients undergoing deep sedation managed by an anaesthesiologist.*

***Non-Anaesthesiologist Administration of Propofol (scenario 2)****: Low-risk patients undergoing moderate sedation managed by the endoscopist, without the involvement of the anaesthesiologist.*

***Savings****: Reduction in staff workload (days) resulting from the adoption of scenario 2 instead of scenario 1 at IRCCS San Raffaele Scientific Institute, ATS Milan, Lombardy Region, and the Italian National Health System (NHS).*
